# Deep Rooting *In-Situ* Expansion of mtDNA Haplogroup R8 in South Asia

**DOI:** 10.1371/journal.pone.0006545

**Published:** 2009-08-07

**Authors:** Kumarasamy Thangaraj, Amrita Nandan, Vishwas Sharma, Varun Kumar Sharma, Muthukrishnan Eaaswarkhanth, Pradeep Kumar Patra, Sandhya Singh, Sashi Rekha, Monika Dua, Narendra Verma, Alla G. Reddy, Lalji Singh

**Affiliations:** 1 Centre for Cellular and Molecular Biology, Hyderabad, India; 2 Department of Biochemistry, Pt. J.N.M. Medical College, Raipur, Chattisgarh, India; University of Hyderabad, India

## Abstract

**Background:**

The phylogeny of the indigenous Indian-specific mitochondrial DNA (mtDNA) haplogroups have been determined and refined in previous reports. Similar to mtDNA superhaplogroups M and N, a profusion of reports are also available for superhaplogroup R. However, there is a dearth of information on South Asian subhaplogroups in particular, including R8. Therefore, we ought to access the genealogy and pre-historic expansion of haplogroup R8 which is considered one of the autochthonous lineages of South Asia.

**Methodology/Principal Findings:**

Upon screening the mtDNA of 5,836 individuals belonging to 104 distinct ethnic populations of the Indian subcontinent, we found 54 individuals with the HVS-I motif that defines the R8 haplogroup. Complete mtDNA sequencing of these 54 individuals revealed two deep-rooted subclades: R8a and R8b. Furthermore, these subclades split into several fine subclades. An isofrequency contour map detected the highest frequency of R8 in the state of Orissa. Spearman's rank correlation analysis suggests significant correlation of R8 occurrence with geography.

**Conclusions/Significance:**

The coalescent age of newly-characterized subclades of R8, R8a (15.4±7.2 Kya) and R8b (25.7±10.2 Kya) indicates that the initial maternal colonization of this haplogroup occurred during the middle and upper Paleolithic period, roughly around 40 to 45 Kya. These results signify that the southern part of Orissa currently inhabited by Munda speakers is likely the origin of these autochthonous maternal deep-rooted haplogroups. Our high-resolution study on the genesis of R8 haplogroup provides ample evidence of its deep-rooted ancestry among the Orissa (Austro-Asiatic) tribes.

## Introduction

India is a melting pot of multi-lingual populations with a unique complex genome diversity [1]. The linguistic diversity prevalent among Indian populations is associated with the presence of four linguistic families: Dravidian (DR), Indo-European (IE), Austro-Asiatic (AA) and Tibeto-Burman (TB) [1]. Of these four groups, AA tribes are considered to be the first settlers of the Indian subcontinent, representing about 30 endogamous tribal populations [2]. The AA linguistic family is traditionally divided into two basic subfamilies: Mon-Khmer and Mundari [3]. Among these two subfamilies, Mundari speakers, the traditional hunter-gatherers, are exclusively found in the Indian subcontinent [3]–[4]. Because Mundari populations are considered to be the earliest inhabitants of the Indian subcontinent, their migration during demic expansion of the agriculturalists in the Neolithic era, as has been suggested for Mon-Khmer speaking Nicobarese [5], appears doubtful.

Numerous studies employing evolutionary-informative markers have demonstrated the origin of various linguistic populations in India [4]–[13]. The phylogeny of Indian mitochondrial DNA (mtDNA) is characterized predominantly by several indigenous haplogroups dispersed exclusively throughout the Indian subcontinent and partially by West Eurasian lineages [8]–[13]. The autochthonous mtDNA haplogroups in Indian populations include: U2a-c R5-8, R30, R31, N1d and N5 in haplogroup N and M2-M6, M30-50 in haplogroup M [6]–[13]. Among the two founder haplogroups, M and N, the former is more prevalent than the latter in the Indian populace [8], [11]. An extrapolation of studies on the N haplogroup led to the discovery of R and several other haplogroups such as U2a-c, R5-R8, R30, R31, N1d and N5 [3], [8]–[9]. It has been estimated that the first footprint of haplogroup R in India took place ∼65Kya and is known as the third most frequent haplogroup, encompassing 11% of the total haplogroups in India after M and N [8]–[9]. Significantly, haplogroups R6 and R7 are more frequent among AA speakers than among other linguistic groups [3].

Though numerous studies have been carried out on the phylogenetic characterization of haplogroup R, there is a dearth of research on its subhaplogroups. To the best of our knowledge, only eight complete mtDNA sequences of haplogroup R8 are available in the database [3], [9]. Therefore, we aim to more accurately trace the genealogy and pre-historic expansion of haplogroup R8 into the Indian subcontinent.

## Results

**Figure 1:**
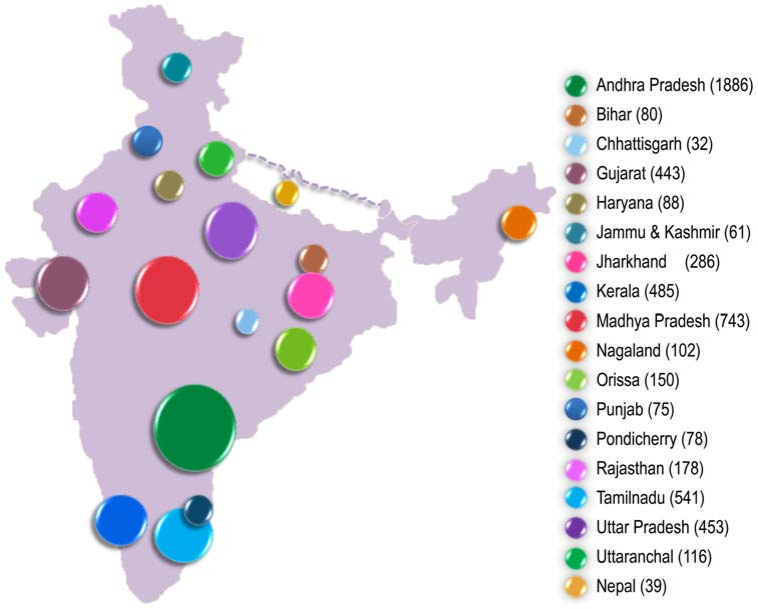
Map of India presenting the total number of samples screened from different states.

**Figure 2:**
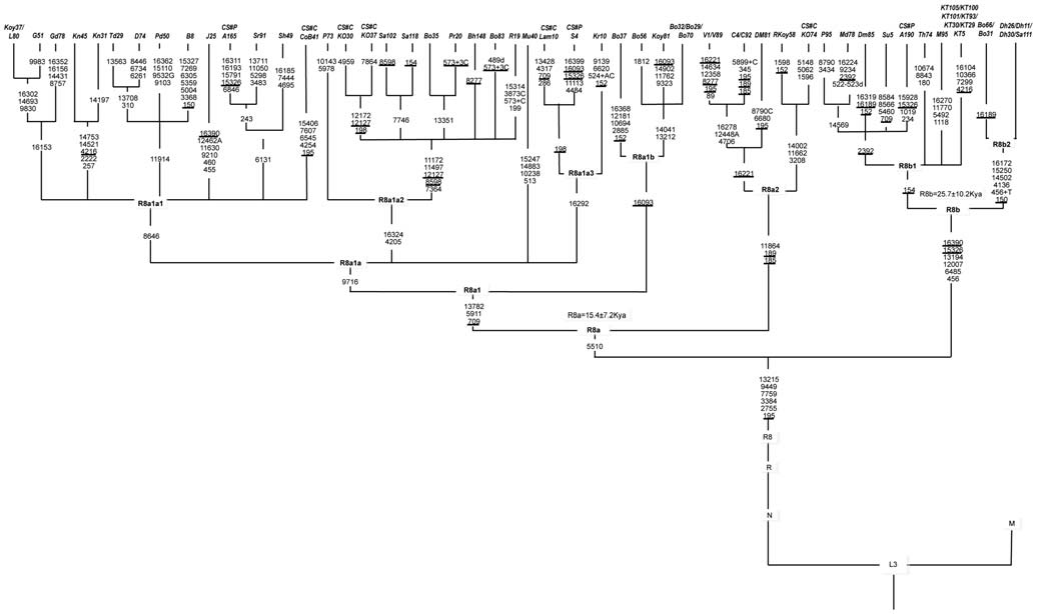
Phylogenetic tree of 54 complete mtDNA sequences. Mutations are scored relative to the rCRS [16]. Additional sequences were taken from the literature and referred by symbols CS#P and CS#C [3], [9]. Suffixes A, C, G and T indicate transversions; “d” denotes deletion and plus sign (+) denotes an insertion; recurrent mutations are underlined; since the variation at 16519 is extremely hypervariable and so not shown here.

**Figure 3:**
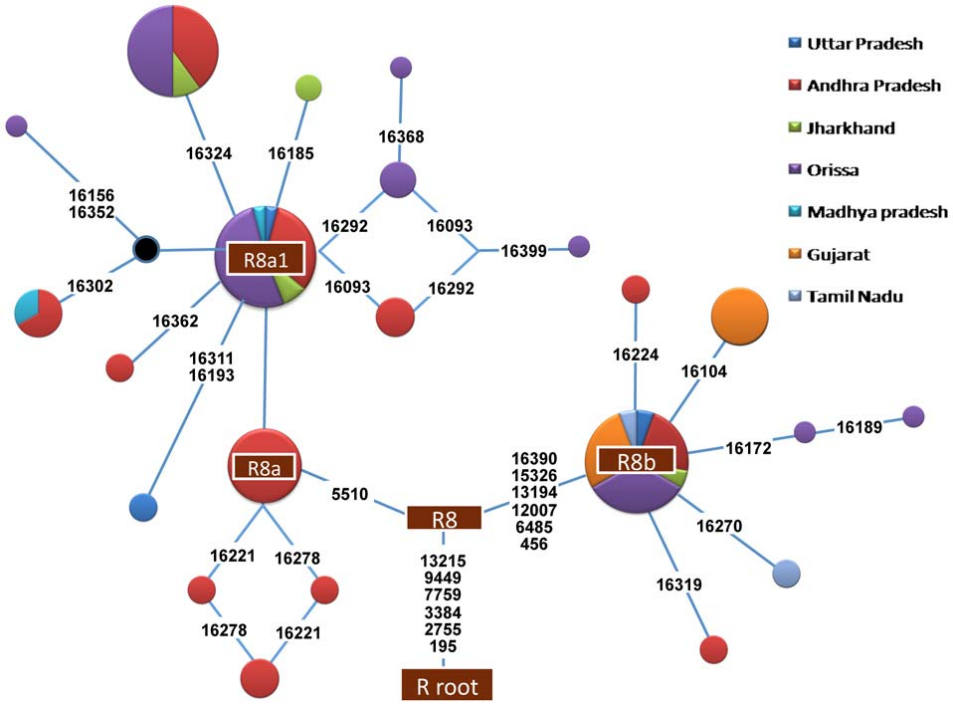
The median joining network of 54 mtDNAs belonging to haplogroup R8. Circle sizes are proportional to the number of mtDNAs with that haplotype.

**Figure 4:**
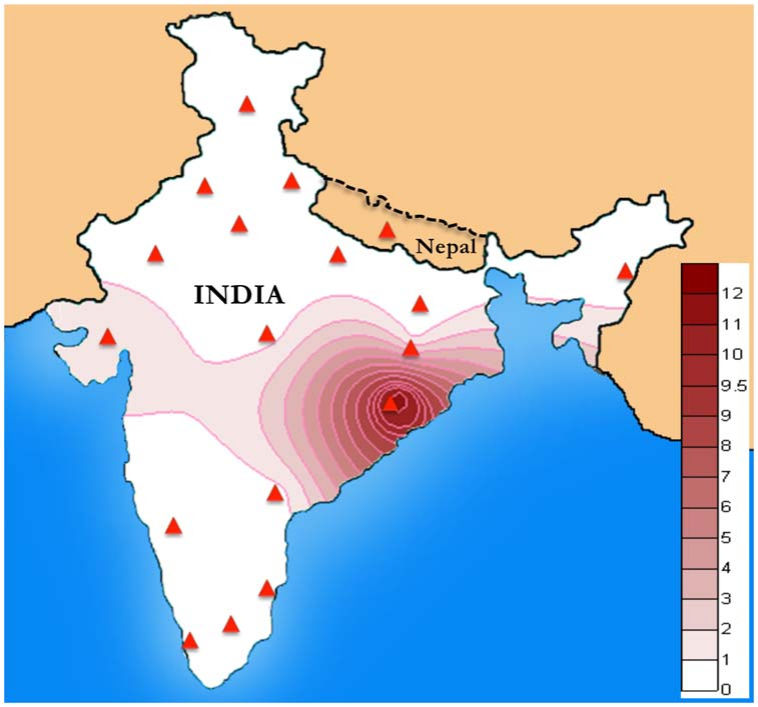
Isofrequency map of mtDNA haplogroup R8. The color gradient demonstrates the frequency in % whereas the points describe the region taken in the study.

We analyzed a total of 5,836 samples from 104 populations across the Indian subcontinent (Figure 1) and identified 54 samples containing haplogroup R8 (Figure 2 & 3). The R8 haplogroup is defined by 13215-9449-7759-3384-2755 sites in the coding regions and single site (195) in the control region. Those HVS-I motifs of Indian populations previously defined as West Eurasian haplogroup H, when matched with revised Cambridge Reference Sequences (rCRS) [6], [13] are now redefined as haplogroup R8. The topology of the previously characterized R8 samples A165, A190, S4, [9] and recently classified Ko74, CoB41, Ko30, Ko37 and Lam10 samples [3] deviates significantly with our samples. A190 [9] grouped with our samples of Panika, Mudiraj, Dommari and Sugali, whereas S4 grouped with Lam10. Upon complete sequencing of the 54 samples, we identified 9 novel sub-haplogroups of haplogroup R8. The coalescent age for haplogroup R8 is 41.7±7.3 Kya while for the two subclades R8a and R8b are 15.4±7.2 Kya and 25.7±10.2 Kya, respectively (Figure 2). R8a is characterized by the motif 709-5510-13782, whereas R8b is characterized by 16390-15326-13194-12007-6485-456. One of the most diverse subclades of R8a, R8a1a1, is separated from the other R8a subclades by a transition at 8646 position. Both of these subclades split into various fine branches (Figure 2). Most R8 subclades are present predominantly in members of the AA language family. The spatial autocorrelation analysis revealed that the highest frequency of this haplogroup occurred towards East India, especially within Orissa (12%) (Figure 4), whereas low frequencies occurred in the Gujarat (1.8%), Madhya Pradesh (0.53%), Uttar Pradesh (0.22%), Andhra Pradesh (0.9%), Chhattisgarh (6%), Jharkhand (1.04%) and Tamil Nadu (0.18%) populations (Figure 4). The Spearman's rank correlation analysis demonstrated a significant correlation between R8 haplogroup frequency and latitude and longitude with r = −0.398 and 0.241 (*p*<0.05), respectively.

HVS-I sequences of the individuals within the R8 haplogroup and who belonged to 30 different ethnic populations, were subjected to estimate intra-population diversity. The diversity indices and neutrality test values are presented in Table 1. The Tajima's and Fu's F_s_ values showed significantly negative values in 18 and 26 populations, respectively (Table 1). Most of the populations showed similar sequence diversity values ranging from 0.8995 (0.051) in Malayan to 0.9940 (0.009) in Kanwar. Orissa populations showed relatively higher values than other populations: Savara 0.9810 (0.022), Bhumia 0.9708 (0.027), Gadaba 0.9667 (0.035), Dhurva 0.9619 (0.039) and Bonda 0.9631 (0.023). A similar trend was also observed in the mean number of pairwise differences: Savara 6.561 (3.23), Bhumia 6.269 (3.11), Gadaba 4.808 (2.47), Dhurva 4.933 (2.54) and Bonda 4.837 (2.43).

**Table 1:** Diversity Indices and Neutrality Tests for the Populations with R8 haplogroup, based on HVS-I sequences.

Population	n	K(%)	S^d^	SD(SE)	Pi	*θ*k (95%)	Tajma'SD(P)f	Fu's Fs (P)f
Bhumia	20	15(75)	32	0.9708 (0.0273)	6.269 (3.112)	30.88(12.14–83.86)	−1.25(−1.109)	−5.74
Chenchu	97	57(59)	62	0.9794 (0.0056)	4.616(2.285)	57.08(38.05–85.91)	−1.99	−25.63
Dhurva	15	12(80)	25	0.9619 (0.039)	4.933(2.544)	25.68(9.10–78.00)	−1.45(−0.071)	−4.99
Dommari	80	52(65)	59	0.9601(0.015)	3.708(1.89)	63.30(40.33–100.24)	−2.26	−26.06
Dusadh	77	33(43)	43	0.9651 (0.007)	5.147(2.52)	21.34(13.40–33.70)	−1.33(−0.087)	−16.13
Gadaba	16	13(81)	29	0.9667 (0.035)	4.808(2.477)	30.01(10.75–90.51)	−1.85	−6
Gond	60	46(76)	70	0.9887 (0.005)	6.671(3.192)	88.42(50.67–158.73)	−1.9	−25.11
Kanwar	32	29(90)	62	0.9940 (0.009)	8.917(4.21)	144.67(56.00–415.50)	−1.45(−0.068)	−19.37
Korku	37	28(75)	43	0.9775 (0.013)	6.366(3.08)	50.73(25.63–104.23)	−1.36(−0.084)	−17.49
Koya	37	25(67)	41	0.9715 (0.013)	5.276(2.60)	32.62(17.08–63.60)	−1.65	−14.41
Malayan	28	18(64)	35	0.8995 (0.051)	3.896(2.01)	20.69(10.04– 43.44)	−2.09	−9.66
Mudiraj	79	61(77)	67	0.9880 (0.005)	5.492(2.67)	120.96(73.75–203.17)	−1.97	−25.37
Munda	29	15(52)	30	0.9655 (0.012)	6.004 (2.94)	11.79(5.80–23.89)	−0.77(−0.235)	−2.57(.146)
Mutharsui	38	32(84)	44	0.9872 (0.010)	4.012(2.05)	92.86(43.64–210.72)	−2.19	−25.85
Padmashal	52	39(75)	49	0.9864 (0.007)	4.843(2.40)	69.21(38.60–127.64)	−1.89	−25.59
Panika	87	57(65)	63	0.9821 (0.006)	5.778(2.79)	70.71(45.80–110.15)	−1.75	−25.27
Rajkoya	62	42(68)	47	0.9841 (0.006)	5.276(2.58)	55.98(33.52–94.84)	−1.58	−25.46
Reddy	30	24(80)	46	0.9793 (0.016)	5.287(2.62)	53.53(24.48– 123.85)	−2.02	−16.77
Savara	21	18(86)	37	0.9810 (0.022)	6.561(3.23)	56.67(21.08–166.84)	−1.42(−0.075)	−9.31
Sonr	96	40(77)	52	0.9594 (0.009)	6.558(3.12)	25.22(16.55–38.16)	−1.12(−0.132)	−17.78
Thoti	30	12(40)	34	0.9218 (0.025)	6.287(3.06)	6.91(3.32–14.06)	−0.97(−0.176)	−0.06(.512)
Bonda	29	21(72)	37	0.9631(0.023)	4.837(2.43)	32.71(15.61–70.95)	−1.79	−12.3
Lambadi	55	49(89)	61	0.9960(0.004)	5.296(2.59)	211.85(102.56–470.83)	−2.07	−25.45
Valmiki	53	35(66)	51	0.9819(0.007)	5.20(2.55)	43.76(25.33–76.65)	−1.84	−25.19
Tadvi	35	25(71)	32	0.9714(0.016)	4.349(2.20)	37.70(19.16–76.36)	−1.55	−18.28
Juang	48	24(50)	34	0.9001(0.034)	5.743(2.8)	18.43(10.47–32.33)	−0.84(−0.225)	−8.28
Bharia	35	22(63)	36	0.9323(0.030)	4.793(2.39)	24.37(12.69–47.45)	−1.61	−11.3
Sugali	21	11(52)	24	0.9238(0.036)	4.590(2.34)	8.61(3.80–19.44)	−1.18(−0.126)	−1.83(.2)
Kotwalia	91	24(26)	44	0.9399(0.009)	5.69(2.75)	10.29(6.28–16.53)	−1.08(−0.134)	−3.83(.119)
Pardhan	42	32(76)	63	0.9861(0.008)	6.55(3.16)	59.62(31.15–118.21)	−1.97	−21.96

n = Sample size; SD = Sequence Diversity; K = Number of different haplotypes and % of sample.

S^d^ = Number of segregating sites; Pi = Average number of Pairwise difference;

f = All p values <.05 for Tajima's and Fu's F_s_ except for where noted.

**Figure 5:**
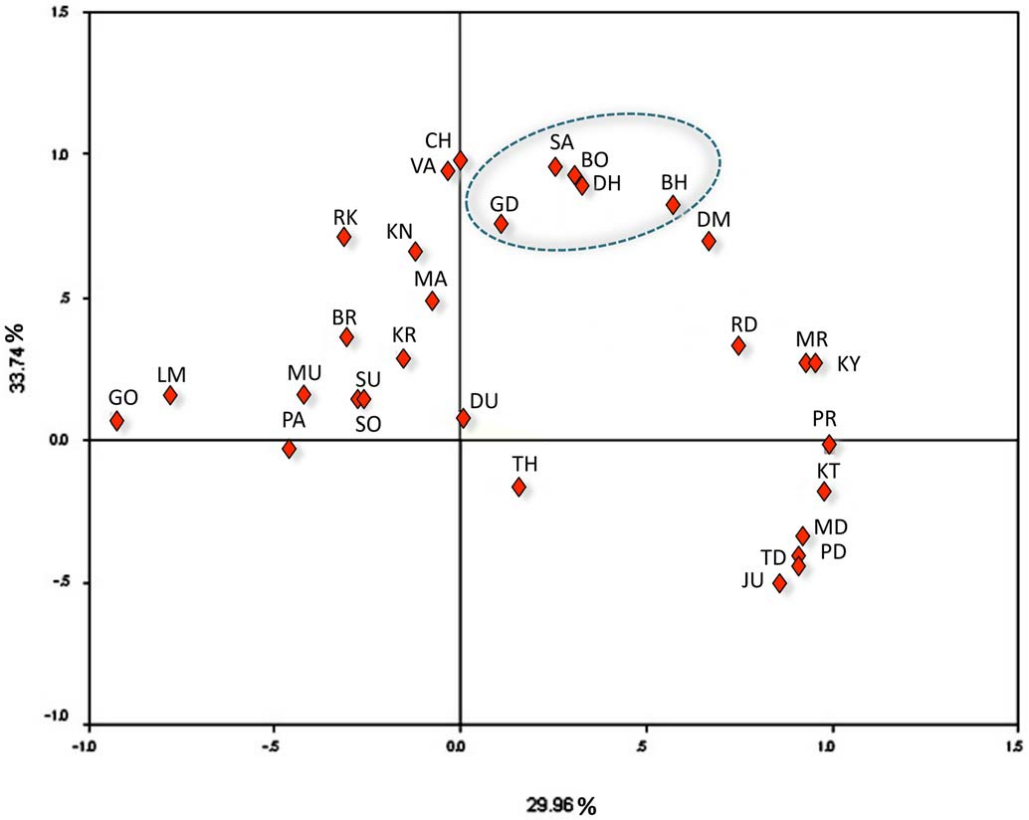
Principal component (PC) analysis of 30 populations from Indian subcontinent which shows the presence of R8. This map accounts 63.70% of the genetic variation. Population codes: GO = gond, LM = lambadi, PA = panika, MU = munda, SU = sugali, BR = bharia, KR = korku, DU = dusadh, MA = Malayan, KN = kanwar, RK = rajkoya, CH = chenchu, VA = valmiki, GD = gadaba, SA = savara, BO = bonda, DH = dhurva, BH = bhumia, DO = dommari, RD = reddy, MR = muttarasui, KY = koya, KT = kotwalia, MD = mudiraj, TD = tadvi, PD = padmashali, JU = juang, PR = pardhan, TH = thoti, SO = sonr.

We have carried out principal component analysis (PCA) to explore the affinities among the populations possessing haplogroup R8, based on the frequency distributions. The PCA plot identified close affinities among the Orissa tribes belonging to the Austro-Asiatic linguistic family (Figure 5). Combined, PC1 and PC2 accounted for a 63.70% variance in the data.

## Discussion

High-resolution analysis of the R8 haplogroup in a total of 5,836 HVS-I (16000–16400) and 54 complete mtDNA sequences characterized two subclades: R8a and R8b. We have further refined these subclades into several subhaplogroups (R8a1, R8a1a1, R8a1a2, R8a1a3, R8a1b, R8a2, R8b, R8b1 and R8b2) based on 38 novel R8 sequences.

Existence of a comparatively high frequency of R8 in Orissa populations, especially among the AA-speaking Mundari tribes, strongly suggests that this haplogroup might have originated among the maternal ancestors of the contemporary AA speakers of the region. To substantiate this hypothesis, we estimated the coalescence time and corroborated with archeological evidence. The time for most recent common ancestors (TMRCA) of R8 (41.7±7.3 Kya) and its subclades R8a (15.4±7.2 Kya) and R8b (25.7±10.2 Kya) divulges the ancient demographic history of this haplogroup (Figure 2).

This haplogroup (R8) is also present in low frequency among the Dravidian and Indo-European speaking family, which can be explained by a language shift or local admixture with the AA-speaking family. Interestingly, this haplogroup was not found in any of the Tibeto-Burman populations analyzed in the present study.

A contour map of the R8 haplogroup revealed its distribution in different geographical regions (Figure 4). It is quite evident from the map that the frequency of this haplogroup is concentrated towards Orissa, Gujarat, Chattisgarh and Jharkhand with highest frequency in Orissa (12%). The Spearman's rank correlation analysis demonstrated a significant correlation of R8 haplogroup frequency to latitude and longitude (*p*<0.05), strong evidence for the relation of genes and geography to this group.

The significant negative values obtained from neutrality tests support the hypothesis of population growth. The PCA plot (Figure 5) found close affinities among the Orissa (AA tribe) population, perhaps due to the high frequency and influence of the R8 haplogroup.

High-resolution study on the origin of the R8 haplogroup provides abundant evidence of its deep-rooted ancestry among the Orissa (AA) tribes. The TMRCA estimates revealed that the initial maternal colonization of this haplogroup occurred during the mid-to-late Paleolithic period, roughly 40 to 45 Kya. The significant relation between the genes and geography is attributed by the spatial analysis of this haplogroup. Moreover, the absence of haplogroup R8 and its subhaplogroups among the Tibeto-Burman speaking populations studied implies socio-cultural practices existing among the populations to be the principle factor for genetic demarcation. Thus, the phylogeographic reconstruction of 54 complete mitochondrial sequences containing haplogroup R8 furnished a better understanding of this partially-characterized haplogroup. Our high-resolution analysis again provided a detailed coding region information for proper classification of a sample, especially in the case of the South Asian haplogroups, which contain several deep-rooted lineages sharing identical coding region mutations with the exception of the HVS-I [14]–[15].

## Materials and Methods

### Ethics Statement

All DNA samples analyzed in the present study were derived from blood samples collected with informed written consent according to protocols approved by the Institutional Ethical Committee of CCMB, Hyderabad.

The samples used in this study were obtained from the DNA bank of CCMB. We have screened a total of 5,836 individuals belonging to 104 ethnic populations from 17 states of India (see Figure 1; Supplementary information Table S1), initially for HVS-I (16000 to 16400) followed by nucleotide position at 3384. Among the 5,836 mtDNA screened, 54 were found to contain basal mutations 13215-9449-7759-3384-2755 which define haplogroup R8. 24 sets of primers were used in sequencing the complete mtDNA. Sequencing of PCR amplicons was performed using the BigDye terminator cycle sequencing kit and ABI 3730XL DNA analyzer (Applied Biosystems, Foster City, USA). The sequences were edited and assembled using AutoAssembler (version 1.4) software (Applied Biosystems, Foster City, USA) to obtain a consensus sequence. These sequences were aligned with rCRS and the mutations were noted [16].

NETWORK (version 4.5) software (www.fluxusengineering.com) was used for phylogenetic reconstruction [17]. The phylogeny obtained was reconfirmed by means of a neighbor-joining tree (1000×bootstrapped) [18], using MEGA (version 4.0) software [19]. We followed the nomenclature system of Richards et al. [20] for reconstructing the phylogenetic tree of haplogroup R8. The isofrequency map for haplogroup R8 was constructed using the Kringing method [21] in the Surfer (version 8.0) program designed by Golden software (Golden Software Inc., Golden, Colorado). Spearman's Rank correlation coefficients between mtDNA haplogroup frequency and latitude and longitude were calculated in StatistiXL (version 1.8) software (StatistiXL, Nedlands, Western Australia) with a *p-*value<0.05 considered statistically significant.

Principal Component (PC) analysis of R5-R8, R30 and R31 lineages in different Indian populations was performed using SPSS (version 11) software (SPSS Inc., Chicago, IL, USA) with mtDNA haplogroup frequencies as an input vector. Coalescence time was calculated using sequence positions between nucleotides 577 to 16023 considering one base substitution per 5,140 years, excluding insertions and deletions [22]. Standard deviation of the rho (σ) estimate was calculated based on Saillard et al. [23]. Descriptive statistical indices and Neutrality tests (Tajima's D, Fu's F_s_) for HVS-I sequences were calculated using Arlequin (version 2.0) software [24]. Complete mtDNA genome sequences generated in this study were submitted to GeneBank (accession numbers FJ467940–FJ467993).

## Supporting Information

Table S1 List of the caste and tribal population studied.
(0.03 MB XLS)
